# Identification of non-small cell lung cancer with chronic obstructive pulmonary disease using clinical symptoms and routine examination: a retrospective study

**DOI:** 10.3389/fonc.2023.1158948

**Published:** 2023-07-28

**Authors:** Bing Zhuan, Hong-Hong Ma, Bo-Chao Zhang, Ping Li, Xi Wang, Qun Yuan, Zhao Yang, Jun Xie

**Affiliations:** ^1^ Department of Respiratory Medicine, Ningxia Hui Autonomous Region People’s Hospital, Yinchuan, Ningxia, China; ^2^ Department of Respiratory Medicine, Ningxia Hui Autonomous Region People’s Hospital Affiliated to Ningxia Medical University, Yinchuan, Ningxia, China; ^3^ School of Biomedical Engineering (Suzhou), Division of Life Sciences and Medicine, University of Science and Technology of China, Suzhou, Jiangsu, China; ^4^ Suzhou Institute of Biomedical Engineering and Technology, Chinese Academy of Sciences, Suzhou, Jiangsu, China; ^5^ Department of Respiratory Medicine, Affiliated Suzhou Science and Technology Town Hospital of Nanjing Medical University, Suzhou, Jiangsu, China; ^6^ Department of Thoracic Surgery, Affiliated Suzhou Science and Technology Town Hospital of Nanjing Medical University, Suzhou, Jiangsu, China

**Keywords:** NSCLC, COPD, machine learning, identification, detection, pulmonary function, emphysema

## Abstract

**Background:**

Patients with non-small cell lung cancer (NSCLC) and patients with NSCLC combined with chronic obstructive pulmonary disease (COPD) have similar physiological conditions in early stages, and the latter have shorter survival times and higher mortality rates. The purpose of this study was to develop and compare machine learning models to identify future diagnoses of COPD combined with NSCLC patients based on the patient’s disease and routine clinical data.

**Methods:**

Data were obtained from 237 patients with COPD combined with NSCLC as well as NSCLC admitted to Ningxia Hui Autonomous Region People’s Hospital from October 2013 to July 2022. Six machine learning algorithms (K-nearest neighbor, logistic regression, eXtreme gradient boosting, support vector machine, naïve Bayes, and artificial neural network) were used to develop prediction models for NSCLC combined with COPD. Sensitivity, specificity, positive predictive value, negative predictive value, accuracy, F1 score, Mathews correlation coefficient (MCC), Kappa, area under the receiver operating characteristic curve (AUROC)and area under the precision-recall curve (AUPRC) were used as performance indicators to evaluate the performance of the models.

**Results:**

135 patients with NSCLC combined with COPD, 102 patients with NSCLC were included in the study. The results showed that pulmonary function and emphysema were important risk factors and that the support vector machine-based identification model showed optimal performance with accuracy:0.946, recall:0.940, specificity:0.955, precision:0.972, npv:0.920, F1 score:0.954, MCC:0.893, Kappa:0.888, AUROC:0.975, AUPRC:0.987.

**Conclusion:**

The use of machine learning tools combining clinical symptoms and routine examination data features is suitable for identifying the risk of concurrent NSCLC in COPD patients.

## 1 Introduction

Lung cancer is a multifactorial disease and is the most common cancer and the leading cause of cancer deaths worldwide (1, 2). Chronic obstructive pulmonary disease (COPD) is a common disease characterized by persistent deterioration of pulmonary function and is a common comorbidity in lung cancer. Relevant studies have shown that the prevalence of COPD is as high as 40%-70% among lung cancer patients (3, 4). Given that both diseases present with decreased pulmonary function and that lung cancer and COPD share common genetic and environmental predisposing factors, numerous clinical studies have confirmed that COPD is a risk factor for the development of lung cancer independently of smoking, and that lung cancer is the most important cause of death in COPD patients (5, 6).

In terms of patient prognosis, clinical studies have shown that lung cancer patients with comorbid COPD have shorter survival times and lower quality of life than lung cancer patients without comorbid COPD, and have a higher risk of prognostic side effects, even life-threatening (7, 8). In addition, COPD and lung cancer are independently and closely related to each other, with similar respiratory symptoms and inflammation that can easily mask the manifestations of lung cancer (9, 10), and patients’ early symptoms and sensations are usually scattered and not easily detected, further leading to delays in seeking medical care and misdiagnosis by physicians (11, 12). How to better differentiate NSCLC patients with COPD is a critical issue that needs to be addressed.

Although the comorbidity of COPD with lung cancer complicates the physiology of patients, many techniques and risk models have been developed to predict and identify the incidence of lung cancer (13, 14). Annual low-dose computed tomography (LDCT) screening is a viable screening tool for early detection of lung cancer or COPD. However, LDCT only targets specific risk groups and unnecessary LDCT in a wide range of patients results in a waste of healthcare resources (15, 16). In addition, imaging changes in chronic pulmonary emphysema do not directly identify the presence of COPD and are ineffective in differentiating COPD from NSCLC when patients are coinfected. Similarly, studies (17, 18) have demonstrated that CT alone is not a reliable clinical endpoint for determining the success of screening for lung cancer or COPD, which has a high false-positive rate and is susceptible to pre-pathological bias in patients.

In terms of risk models, early models were relatively simple and included recognized risk factors such as gender, age, and smoking history (19, 20), while traditional standard statistical risk models were not as effective in distinguishing and assessing lung cancer (21, 22). Recently, artificial intelligence and machine learning methods have played a great role in improving risk prediction (23, 24). Compared with traditional models, accurate prediction and recognition can be generated by selecting prediction factors and corresponding models, which can distinguish disease types that are difficult to distinguish, and require less restrictive assumptions. At the same time, it has greater flexibility in dealing with the non-linear relationship between missing values and parameters.

In this study, we focused our analysis on non-small cell lung cancer (NSCLC) with COPD, as NSCLC accounts for approximately 85% of lung cancer cases worldwide (25). To investigate the clinical characteristics of NSCLC combined with COPD, this study evaluated six different recognition classification techniques: K-nearest neighbor (KNN), Logistic regression (LR), eXtreme gradient boosting (XGB), Support vector machine (SVM), Naïve Bayes (NB), and Multilayer perceptron (MLP). Each of the above algorithms represents different operational properties (26)and is widely used by other researchers (27, 28).

In this study, the clinical characteristics of patients with NSCLC combined with COPD were retrospectively analyzed in the current research progress, and multiple machine learning methods were used to develop an optimal prediction model for NSCLC combined with COPD using clinical symptoms and routine examination data from patients’ electronic health records, to establish the optimal set of characteristics in order to identify patients with NSCLC before clinical diagnosis, and to screen important clinical features of patients with NSCLC combined with COPD to improve the survival rate of patients at the early identification stage.

The main contributions of this study are as follows.

1. To investigate suitable machine learning methods for the identification of COPD combined with NSCLC in six supervised classification models (i.e., KNN, LR, XGB, SVM, NB and MLP).
2. To build a comprehensive feature set of patients’ clinical conditions, demographics, pulmonary function parameters, lung CT parameters, biomarkers, blood gas analysis and cancer information to find the optimal feature set and robust indicators for NSCLC combined with COPD identification task.

## 2 Methods

### 2.1 Study population

This was a single-center, retrospective analysis which did not involve patient safety or privacy, and an ethical exemption was granted. In this study, a total of 237 patients with NSCLC combined with COPD and NSCLC admitted to the People’s Hospital of Ningxia Hui Autonomous Region from October 2013 to July 2022 were collected, and clinical data including gender, age, BMI, smoking history, COPD history, major symptoms, pathological type, cancer stage, pulmonary function parameters, emphysema volume, airway wall thickness, and tumor markers were collected from both groups to construct a characteristic dataset of patients with NSCLC combined with COPD. Among them, clinical symptoms were classified according to the literature (29).

The inclusion criteria for this study were as follows. The diagnosis of COPD conforms to the diagnostic criteria of the 2022 global strategy for prevention, diagnosis and management of COPD (30), that is, patients with dyspnea, chronic cough or expectoration, history of recurrent lower respiratory tract infection, or history of exposure to risk factors of the disease, after inhaling bronchodilators, forced expiratory volume in one second (FEV1)/forced vital capacity (FVC)<0.70.

The diagnostic criteria for NSCLC are pathological evidence, which is classified as squamous cell carcinoma, adenocarcinoma, and large cell carcinoma according to the type of pathological histology; the TNM staging of lung cancer is in accordance with the 8th edition of TNM staging of lung cancer issued by the International Society for the Study of Lung Cancer in 2015 (31).

The exclusion criteria for this study were as follows. ① Excluding patients with other lung diseases other than COPD and NSCLC; ② Patients with primary tumors at other sites; ③ Patients with previous or current connective tissue disease and blood disease; ④ Patients currently suffering from severe infection; ⑤ Patients who cannot co-operate with pulmonary function test.

### 2.2 Data selection

All patients in this study underwent pulmonary function tests, completed chest CT scans and were scored according to Goddard semi-quantitative CT emphysema, which was analyzed and measured automatically using Philips IntelliSpace Portal V9 COPD software, in addition to laboratory tests for biomarkers and routine blood analysis of the patients. In this study, the characteristics were divided into six areas, patient baseline characteristics, pulmonary function parameters, lung CT parameters, biomarkers, routine blood information, and lung cancer information. In this study, the clinical symptoms of the patients were taken into consideration and the corresponding symptoms were classified and divided. Specific classification information is shown in **Supplementary Tables 1**, **2**.

### 2.3 Data pre-processing and feature engineering

Forty input features and one output feature (confirmed clinical diagnosis) were extracted from the EMRs and described in detail in the Results section (see **Table 1**). Further processing of the missing values was required according to clinical needs. During the processing of missing values, the missing discrete variables were replaced with the most frequent category associated with the feature, and for the missing continuous variables, four filling methods were used in this study to compare the processing. The four methods were: filling with zero constant, filling with mean, filling with random forest regression based on zero value, and filling with random forest regression based on mean. The comparison results are shown in **Supplementary Figures 1**, **2**.

**Table 1:** Descriptive statistics analysis.

	Characteristic		NSCLC group (102)	COPD_NSCLC group (135)	P value
Demographic parameters	Gender	Male	55	114	<0.001*
		Female	47	21	
	Age	year	60.50 (9.66)	69.80 (9.82)	<0.001*
	BMI	kg/cm2	24.035 (3.58)	22.86 (4.08)	0.022
	Smoking index	cigarettes*year	120.00 (0.00, 400.00)	600.00 (225.00, 1000.00)	<0.001*
	Treatment method	0	77	86	0.053
		1	25	49	
	Survival status	0	73	117	0.004*
		1	29	18	
Clinical Symptoms	Clinical Symptoms1	0	27	17	0.007*
		1	75	118	
	Clinical Symptoms2	0	51	39	0.001*
		1	51	96	
	Clinical Symptoms3	0	92	112	0.111
		1	10	23	
	Clinical Symptoms4	0	49	51	0.113
		1	53	84	
	Clinical Symptoms5	0	85	110	0.712
		1	17	25	
	Clinical Symptoms6	0	97	134	0.043*
		1	5	1	
	Clinical Symptoms7	0	102	134	0.384
		1	0	1	
	Clinical Symptoms8	0	91	100	0.004*
		1	11	35	
Pulmonary function parameters	FEV1	L	2.19 (1.80, 2.74)	1.61 (1.08, 1.91)	<0.001*
	FEV1/Pred	%	87.00 (75.38, 100.38)	50.10 (43.20, 72.20)	<0.001*
	FVC	L	2.74 (0.80)	2.80 (0.82)	0.598
	FEV1/FVC	%	79.59 (75.23, 83.42)	57.63 (46.36, 64.00)	<0.001*
	RV/TLC	%	51.88 (41.54, 60.37)	56.35 (48.34, 63.60)	0.002*
	DLCO	ml·kPa-1/s	6.66 (6.32, 8.72)	6.66 (4.46, 6.66)	<0.001*
Lung CT Parameters	Goddard		1 (0.00, 3.00)	16 (7.00, 23.00)	<0.001*
	E		14.25 (5.15, 34.24)	30.00 (7.00, 60.00)	0.004*
	EI	%	0.40 (0.10, 1.70)	16.10 (2.40, 25.00)	<0.001*
	Affected side emphysema ratio	%	0.40 (0.1, 1.70)	16.60 (2.00, 26.50)	<0.001*
	Intact side emphysema ratio	%	0.35 (0.10, 1.25)	3.70 (0.90, 6.70)	<0.001*
	WA	%	47.72 (41.60, 53.15)	46.10 (40.90, 53.90)	0.956
Biomarkers	CEA	ng/ml	2.58 (1.30, 17.65)	3.25 (1.88, 10.98)	0.274
	CA125	U/ml	14.54 (9.54, 38.825)	24.20 (11.21, 88.82)	0.029*
	NSE	ng/ml	13.54 (11.27, 17.39)	16.66 (12.06, 19.04)	0.008*
	SCC	ng/ml	0.80 (0.60, 1.20)	1.30 (0.80, 2.05)	<0.001*
	CYFRA21-1	ng/ml	2.47 (1.42, 5.50)	4.23 (3.21, 6.12)	<0.001*
Routine blood information	Gran	*10^9/L	3.62 (2.91, 4.96)	4.23 (3.21, 6.12)	0.003*
	LYMPH	*10^9/L	1.53 (1.16, 2.04)	1.47 (1.11, 2.09)	0.775
	PLT	*10^9/L	230.00 (181.00, 273.25)	206.00 (167.00, 265.00)	0.219
	CRP	mg/L	4.75 (1.69, 18.83)	12.00 (3.00, 20.40)	0.018*
	FIB	g/L	3.23 (2.74, 3.88)	3.17 (2.63, 4.19)	0.691
Lung cancer information	TNM	1	25	22	<0.001*
		2	5	5	
		3	4	6	
		4	27	15	
		5	10	17	
		6	2	15	
		7	16	1	
		8	13	36	
		9	0	18	
	Cancer type	0	26	80	<0.001*
		1	76	55	
	Pathological location	0	66	65	0.011*
		1	36	70	

^∗^represents statistically significant; NSCLC, Non-small cell lung cancer; BMI, Body mass index; FEV1, Forced expiratory volume in one second; FEV1/Pred, FEV1% of predicted; FVC, Forced vital capacity; RV, Residual volume; TLC, Total lung capacity; DLCO, Diffusion capacity for carbon monoxide; Goddard, Goddard score; GOLD, Global Initiative for Chronic Obstructive Lung Disease; E, Emphysema score; EI, Emphysema index; WA, Airway wall area percent; CEA, Carcinoembryonic antigen; CA125, Carbohydrate antigen 125; NSE, Neuron-specific enolase; SCC, Squamous cell carcinoma; CYFRA21-1, Cytokeratin 19 fragment; Gran, Neutrophilic granulocyte count; LYMPH, Lymphocyte count; PLT, Platelet count; CRP, C-reactive protein; FIB, Fibrinogen; TNM, Tumor node metastasis.

**Figure 1:**
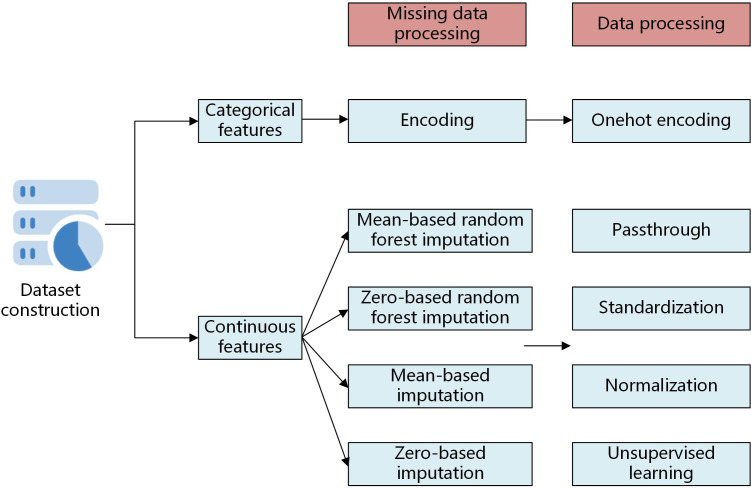
Data processing flow chart.

Among them, random forest interpolation was used to predict and fill the missing values as a test set. These four methods are classical filling methods. After that, considering the existence of extreme values for some features and the unstable data range, the continuous features were standardized, normalized or unsupervised learning processed and the categorical features were one-hot coded. For the processing methods of continuous features, the purpose of standardization is to have similar distribution of data for different features, which of normalization is to compare the differences of features in the same interval, and which of unsupervised processing is to reduce the influence of anomalous values. The data processing flow is shown in **Figure 1**.

### 2.4 Statistical analysis

Based on the information collected in this study on patients’ clinical conditions and routine examinations, data that were normally distributed were expressed as mean (SD), data that were not normally distributed were expressed as median (IQR), as well as for categorical variables were expressed as percentages. A chi-square test was performed for categorical variables. For continuous variables, Student t-test was used based on the assessment of normality of the variables. If the distribution was not normal, the Wilcoxon Mann-Whitney U test was used. When P<0.05, there was statistical significance, which was a suspected factor for both diseases. All statistical analyses were performed using SPSS version 22.0. The results of the specific analyses are shown in **Table 1**.

### 2.5 Model establishment and evaluation

Six commonly used classifier algorithms were chosen to this study, including K-nearest neighbor (KNN), Logistic regression (LR), eXtreme gradient boosting (XGB), Support vector machine (SVM), Naïve Bayes (NB), Multilayer perceptron (MLP). The transformation of features, construction of models, and tuning of hyperparameters were integrated by way of building a machine learning pipeline flow.

This study used the confusion matrix, accuracy, sensitivity (recall), specificity, positive predictive value [PPV (precision)], negative predictive value (NPV), F1 score, MCC, Kappa, area under the receiver operating characteristic curve (AUROC), and the area under the precision-recall curve (AUPRC) to evaluate and compare the comprehensive performance of different models.

**Figure 2:**
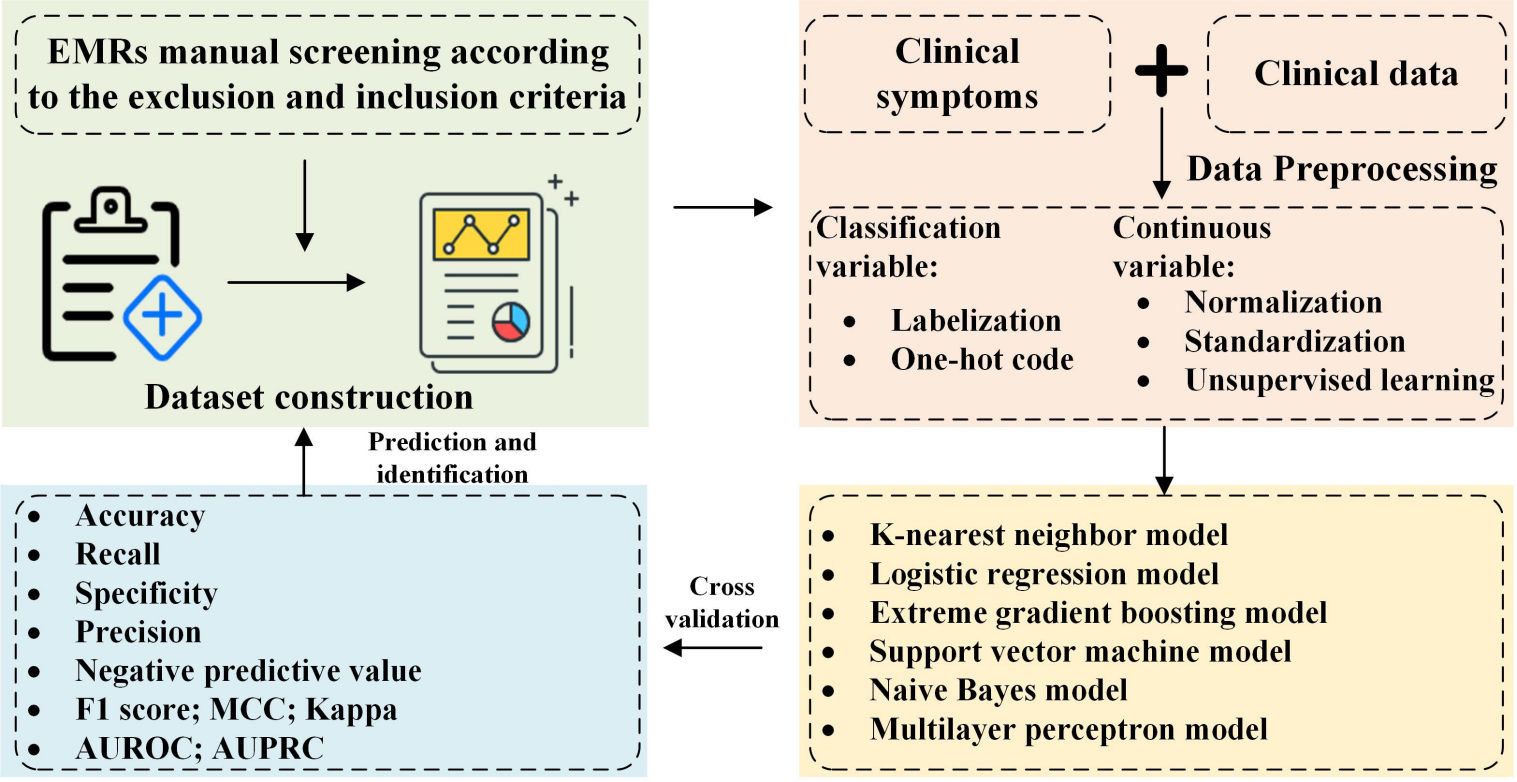
Experimental data analysis process.

The python library ‘Sklearn’ was used to apply the machine learning algorithms for identification (32). Except for hyperparameters mentioned above, the other hyperparameters of each model were set as the default values. Considering the size of dataset was small and to avoid the possible bias, stratified 10-fold cross validation was used to assess the performance of our models: 10 subsets were constructed by dividing the overall dataset randomly, and each subset was set as a testing set and the remaining subsets were set as a training set. The performance of our models was reported as the average across all the 10 testing sets. In machine learning, this is widely used and preferred validation technique, literature (33) had explained the advantages of using such a method in detail. The model building process is shown in **Figure 2**.

## 3 Result

### 3.1 Demographic and pathological characteristics

The NSCLC combined with COPD group and NSCLC group were sorted, including 135 patients with NSCLC combined with COPD and 102 patients with NSCLC. The average age of the former group was 69.80 (9.82), and that of the latter group was 60.72 (9.86). To determine the statistical significance of the two groups of data, univariate statistical analysis was performed on the data.

As shown in **Table 1**, the univariate statistics showed that in the NSCLC combined with COPD group and the NSCLC group, gender, age, BMI, smoking index, treatment method, survival status, clinical symptoms 1, clinical symptoms 2, clinical symptoms 6, clinical symptoms 8, FEV1, FEV1/Pred, FEV1/FVC, RV/TLC, DLCO, Goddard score, E, EI, intact side emphysema ratio, affected side emphysema ratio, CA125, SCC, CYFRA21-1, Gran, CRP, TNM, cancer type, pathological location, significant P<0.05 for the above characteristics, as suspected factors.

### 3.2 Model performance

To identify and predict NSCLC patients and NSCLC combined with COPD patients, this study compared six algorithms based on routine clinical data and patients’ clinical symptoms, and filtered the hyperparameters of each algorithm after performing grid search, and detailed parameter comparisons are shown in **Supplementary Tables 3**-**8**.

In this study, six different machine learning models were built by grid search with various combinations of parameters and using 10-fold cross-validation. Each model was evaluated for accuracy, recall, specificity, precision, NPV, F1 score, MCC, Kappa, AUROC, and AUPRC, and the corresponding means ( ± SD) were calculated. **Table 2** summarizes performance of the different models in the NSCLC combined with COPD group and the NSCLC patient group.

**Table 2:** Performance of different models in the dataset.

	KNN	XGB	LR	SVM	GNB	MLP
Accuracy	0.921 ± 0.061	0.927 ± 0.037	0.939 ± 0.056	0.946 ± 0.032	0.805 ± 0.110	0.939 ± 0.038
Recall	0.919 ± 0.087	0.960 ± 0.049	0.940 ± 0.092	0.940 ± 0.049	0.776 ± 0.132	0.908 ± 0.074
Specificity	0.924 ± 0.100	0.879 ± 0.097	0.938 ± 0.107	0.955 ± 0.069	0.850 ± 0.182	0.986 ± 0.043
Precision	0.954 ± 0.062	0.925 ± 0.053	0.965 ± 0.057	0.972 ± 0.043	0.897 ± 0.114	0.991 ± 0.027
NPV	0.896 ± 0.102	0.942 ± 0.071	0.927 ± 0.105	0.920 ± 0.066	0.729 ± 0.130	0.888 ± 0.081
F1 score	0.932 ± 0.054	0.940 ± 0.029	0.948 ± 0.050	0.954 ± 0.027	0.823 ± 0.102	0.945 ± 0.038
MCC	0.846 ± 0.118	0.852 ± 0.078	0.885 ± 0.100	0.893 ± 0.065	0.626 ± 0.218	0.886 ± 0.069
Kappa	0.837 ± 0.125	0.845 ± 0.082	0.874 ± 0.114	0.888 ± 0.066	0.607 ± 0.223	0.877 ± 0.076
AUROC	0.956 ± 0.050	0.977 ± 0.025	0.976 ± 0.023	0.975 ± 0.032	0.908 ± 0.067	0.941 ± 0.056
AUPRC	0.970 ± 0.036	0.988 ± 0.012	0.985 ± 0.015	0.987 ± 0.016	0.940 ± 0.043	0.954 ± 0.071

From the results, all six models, KNN, XGB, LR, SVM, NB, and MLP, had good performance. The SVM model outperformed the other models in terms of accuracy, F1 score, MCC, and Kappa combined evaluation metrics. In terms of precision and specificity, the MLP model performs best. For recall and NPV, the XGB model performed best. In terms of AUROC, AUPRC, XGB, LR and SVM models performed similarly.

### 3.3 Importance of features on prediction

**Figure 3:**
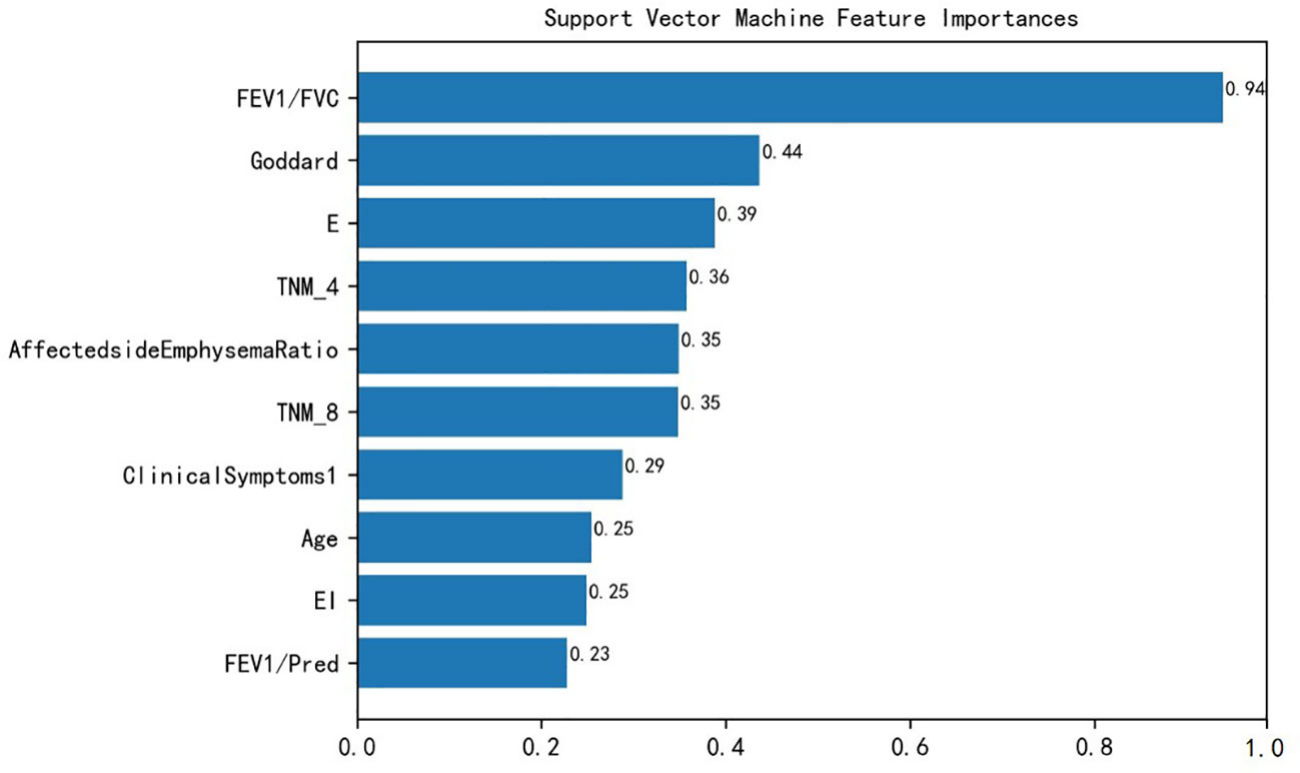
SVM features importance ranking. FEV1/FVC, Forced expiratory volume in one second/forced vital capacity; Goddard, Goddard score; E, Emphysema score; TNM, Tumor node metastasis; Clinical Symptoms1: Irritant cough, dry cough; EI, Emphysema index; FEV1/Pred, FEV1% of predicted.

In this study, the best models were screened to calculate the feature importance. The above results showed that the SVM model performed the best. The SVM model measured the feature importance by kernel function coefficients. **Figure 3** represents the top 10 features importance ranking of SVM, which were FEV1/FVC, Goddard score, E, TNM4, Affected side Emphysema Ratio, TNM8, Clinical Symptoms 1, Age, EI, FEV1/Pred.

## 4 Discussion

Lung cancer as the most important cause of death in COPD patients has been a hot topic in lung disease research. In this study, we first performed statistical analysis of clinical data and patient symptoms and compared possible suspicious factors, established and evaluated six machine learning algorithms for differentiating COPD combined with NSCLC patients, determined the best parameters of the models by grid search, ensured the generalization ability of the models by cross-validation. The performance of each model was evaluated based on several evaluation metrics such as accuracy, sensitivity, specificity, PPV, NPV, F1 score, MCC, Kappa, AUROC, and AUPRC, and the importance of the features in the optimal model was ranked.

By comparing the model performance evaluation results provided in **Table 2**, it is found that the SVM-based model had the best comprehensive ability to identify patients with COPD combined with NSCLC among all the proposed models. An identification model with high sensitivity and specificity could effectively provide decision support for physicians, thus avoiding to some extent the misidentification of NSCLC patients and the overestimation of patients with comorbid COPD.

In recent years, it has been found that patients with a history of COPD have a higher risk of developing lung cancer than those without a history of COPD, and the early diagnosis rate of lung cancer occurring based on COPD is low and survival time is short (34), so finding risk factors for lung cancer based on COPD and predictive indicators affecting prognosis appears to be It is especially important.

In comparing the clinical disease characteristics of the NSCLC patient group and the NSCLC combined with COPD patient group, the comparison of the importance of the features by the SVM model showed that the symptoms from the patients’ irritating cough and dry cough could assist in determining whether the patients were combined with COPD, because COPD itself is a respiratory disease with continuous airflow limitation, and the combined COPD further aggravates the patients’ cough and sputum.

In terms of pulmonary function characteristics, the SVM model showed that the pulmonary function index FEV1/FVC was an important feature to distinguish between the two diseases, and similarly, FEV1/Pred also had the predictive ability to classify comorbidities, and a related study (35) also showed that GOLD grading based on pulmonary function index FEV1/FVC could be used as a predictor to determine the prognosis and survival of patients with NSCLC combined with COPD. In terms of lung cancer information characteristics, the current study found that patients with combined COPD had a higher TNM severity grade than patients with NSCLC, and a study by Papi et al. (36) showed that COPD increased the risk of developing NSCLC in patients with smoking.

In this study, Goddard score was shown to be the second most important feature to distinguish between the two patients. The Goddard score is an assessment method based on imaging phenotypes to assess the severity of emphysema in patients (37). In this study, the Goddard score was much greater in patients with NSCLC combined with COPD than in patients with NSCLC. Similarly, the affected side emphysema ratio, E, and EI could likewise provide help in classifying combined COPD. It has been shown that emphysema is independently associated with COPD morbidity and mortality and that emphysema can help to screen lung cancer patients with comorbid COPD (38). In addition, in terms of demographic baseline, the age of the patients was shown to have a greater impact according to the model, which is in line with Wang et al. (35).

In addition, relevant studies on COPD and NSCLC were compiled and compared. Wang et al. (39) predicted the risk of cancer in the general population by an XGB model, which classified patients into low, medium, and high-risk groups with AUROC of 0.881. Zeng et al. (40) also used the XGB model to predict the severe deterioration of COPD patients in the second year with AUROC of 0.866. In contrast, the present study further defined and narrowed the disease scope by predicting only identifying patients with NSCLC combined with COPD, and provided a more comprehensive and objective basis for the evaluation based on data from patient symptoms and routine examinations. Compared to the Extended Spitz model (19) requires genetic test information, which was not available in routine clinical data. In addition, the EPIC (European Prospective Investigation into Cancer and Nutrition) (41) and HUNT(Helseundersøkelsen iN-ord-Trøndelag) (20) models used smoking status (e.g., smoking duration, smoking intensity, and years since cessation) as predictors and lack support from other aspects of patient clinical data.

Of course, there are some limitations in the present study. First, due to the inclusion and exclusion criteria, our results only applied to specific patients who met these criteria and did not reflect all NSCLC as well as NSCLC combined with COPD populations. For example, the identification ability of the proposed model will be limited if patients have other diseases or comorbidities such as asthma. Second, variable data quality and different clinical symptoms resulted in some clinical features (e.g., WA, etc.) not being significant in this study; on the one hand, these features may lack the ability to distinguish patients with COPD combined with NSCLC, and on the other hand, for example, some continuous features such as DLCO had a slightly larger proportion of missing data. Although we used an imputation strategy for processing, it inevitably changed the original distribution and undermined the objective reasonableness of the data to some extent. Therefore, the correlation results did not represent the actual situation. Third, the patients included in this study were from the same center and the number of patients was a small sample size. Patient data were collected from 2013 to 2022 over a period of almost a decade, with potential patient recall bias that may result from the retrospective approach. External validation was not performed in the study, which may also limit the ability to generalize the methods of this study. Therefore, the applicability to other contexts remains to be determined. Fourth, the method of this study was limited to the six classical machine learning methods mentioned above, and considering the insufficient sample size and the relevant literature (42) showing that deep learning could not improve the prediction performance more significantly. Therefore, deep learning was not compared, but the results of machine learning research were sufficient to verify and compare the characteristics and advantages of different models. In the future, we further expand the dataset by including more patients from multiple centers and using publicly available patient data from other institutions for external validation.

## 5 Conclusions

Our study confirmed that the SVM model was the best model to identify patients with NSCLC combined with COPD and patients with NSCLC. Pulmonary function indicators FEV1/FVC, FEV1/Pred, Goddard score, affected side emphysema ratio, E, and EI were important indicators. In addition, our study also provided a good feature set for identifying patients with NSCLC combined with COPD using machine learning methods. With further testing and development, our proposed method is expected to provide decision support for clinicians and assist them in giving a definitive clinical diagnosis. In the future, more comprehensive inclusion criteria and a dataset containing more cases will be further investigated.

## Data availability statement

The original contributions presented in the study are included in the article/**Supplementary Material**. Further inquiries can be directed to the corresponding authors.

## Ethics statement

The study protocol was approved by the Institutional Review Board of the Ningxia Hui Autonomous Region People's Hospital (IRB no. [2019] Ethics [Scientific Research] 375. 2019-08-16). This study was conducted in accordance with the Declaration of Helsinki. Written informed consent from the patients/participants or patients/participants’ legal guardians/next of kin was not required to participate in this study in accordance with the national legislation and the institutional requirements.

## Author contributions

ZY and JX designed the study. BZ, and HHM contributed to the conception of the study and completed the manuscript together. B-CZ, and PL contributed significantly to statistical analysis and manuscript preparation. XW and QY helped perform the analysis with constructive discussions. All authors contributed to the article and approved the submitted version.
