# Supplementary material for: Identification of non-small cell lung cancer with chronic obstructive pulmonary disease using clinical symptoms and routine examination: a retrospective study
Source: Front Oncol. 2023 Jul 28;13:1158948. doi: 10.3389/fonc.2023.1158948 (PMC10419203; doi:10.3389/fonc.2023.1158948)
Supplement: Supplementary file 1 [file DataSheet_1.docx]

Supplementary Material

Supplementary Table S1. Characteristics of NSCLC combined with COPD group and NSCLC group.

|  |  | **Abbreviation** | **Unit** | **Range** |
| --- | --- | --- | --- | --- |
| Baseline Characteristics | Gender | F/M |  | (M =1, F =2) |
|  | Age | Age | year |  |
|  | Body mass index | BMI | kg/cm2 |  |
|  | Smoking Index | Smoking Index | cigarettes*year |  |
|  | Tumor treatment | Tumor Treatment | 0 | Surgery, chemotherapy, radiotherapy |
|  | Palliative treatment | Palliative Treatment | 1 | Anti-infective support, symptomatic treatment |
|  | Survival status | Survival |  |  |
|  | Clinical symptoms | Clinical symptoms |  |  |
| Pulmonary Function Parameters | Forced expiratory volume in one second | FEV1 | L | 0- |
|  | FEV1% of predicted | FEV1/Pred | % | 0-100 |
|  | Forced vital capacity | FVC | L | 0- |
|  | FEVI/FVC | FEV1/FVC | % | 0-100 |
|  | Residual volume / Total lung capacity | RV/TLC | % | 0-100 |
|  | Diffusion capacity for carbon monoxide | DLCO | ml·kPa-1/s | 0- |
| Lung CT Parameters | Goddard score | Goddard |  | 0-24 |
|  | Emphysema score (E) | E |  |  |
|  | Emphysema index (EI) | EI | % | 0-100 |
|  | Affected side emphysema ratio (%) | Affected side ratio | % | 0-100 |
|  | Intact side emphysema ratio (%) | Intact side ratio | % | 0-100 |
|  | Airway wall area percent | WA | % | 0-100 |
| Biomarker | Carcinoembryonic antigen | CEA | ng/ml | 0-4.7 |
|  | Carbohydrate antigen-125 | CA125 | U/ml | 0-35 |
|  | Neuron-specific enolase | NSE | ng/ml | 0-16.3 |
|  | Squamous cell carcinoma | SCC | ng/ml | 0-1.5 |
|  | Cytokeratin 19 fragment | CYFRA21-1 | ng/ml | 0-2.08 |
| Blood Routine Analysis | Neutrophilic granulocyte count | Gran | *10^9/L | 1.8-6.3 |
|  | Lymphocyte count | LYMPH | *10^9/L | 1.1-3.2 |
|  | Platelet count | PLT | *10^9/L | 125-350 |
|  | C-reactive protein | CRP | mg/L |  |
|  | Fibrinogen | FIB | g/L | 2-4 |
| Lung Cancer Information | Squamous carcinoma | Pathological typing | 0 | 0/1 |
|  | Adenocarcinoma |  | 1 |  |
|  | Location type (Peripheral) | Location Typing | 0 | 0/1 |
|  | Location type (central type) |  | 1 |  |
|  | Tumor node metastasis | TNM |  | 0-9 |

∗ represents statistically significant; NSCLC, Non-small cell lung cancer; BMI, Body mass index; FEV1, Forced expiratory volume in one second; FEV1/Pred, FEV1 % of predicted; FVC, Forced vital capacity; RV, Residual volume; TLC, Total lung capacity; DLCO, Diffusion capacity for carbon monoxide; GOLD, Global Initiative for Chronic Obstructive Lung Disease; E, Emphysema score; EI, Emphysema index; WA, Airway wall area percent; CEA, Carcinoembryonic antigen; CA125, Carbohydrate antigen 125; NSE, Neuron-specific enolase; SCC, Squamous cell carcinoma; CYFRA21-1, Cytokeratin 19 fragment; Gran, Neutrophilic granulocyte count; LYMPH, Lymphocyte count; PLT, Platelet count; CRP, C-reactive protein; FIB, Fibrinogen; TNM, Tumor node metastasis.

Supplementary Table S2. Clinical symptoms classification.

| **Clinical Symptoms** | **Number** |
| --- | --- |
| Irritant cough, dry cough | 1 |
| Expectoration | 2 |
| Bloody sputum, hemoptysis | 3 |
| Chest tightness, shortness of breath, wheezing | 4 |
| Chest pain, shoulder and back pain | 5 |
| Nausea, vomiting, abdominal pain | 6 |
| Hoarse voice | 7 |
| Systemic symptoms | 8 |
| Asymptomatic | 0 |

Systemic symptoms 8 include：

Weakness, neck swelling, emaciation, dizziness, headache, lumbago, acid reflux, palpitations, anorexia, fever, hyperhidrosis, profuse sweating, bilateral lower limb edema, swollen eyelids, itchy throat

| 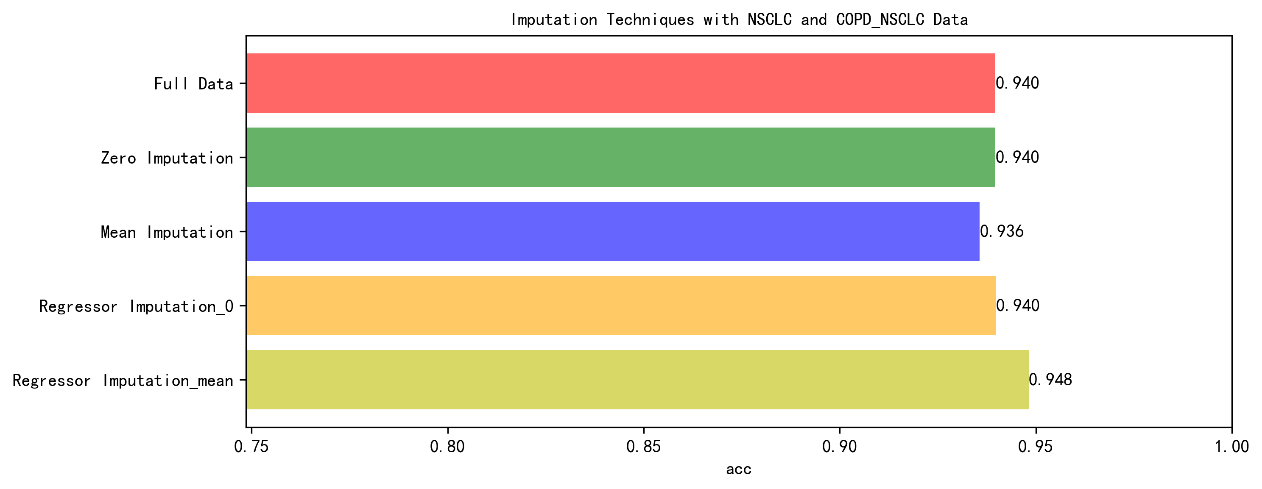 |
| --- |
| **Supplementary Figure S1.** Accuracy comparison of different pre-processing. |
| 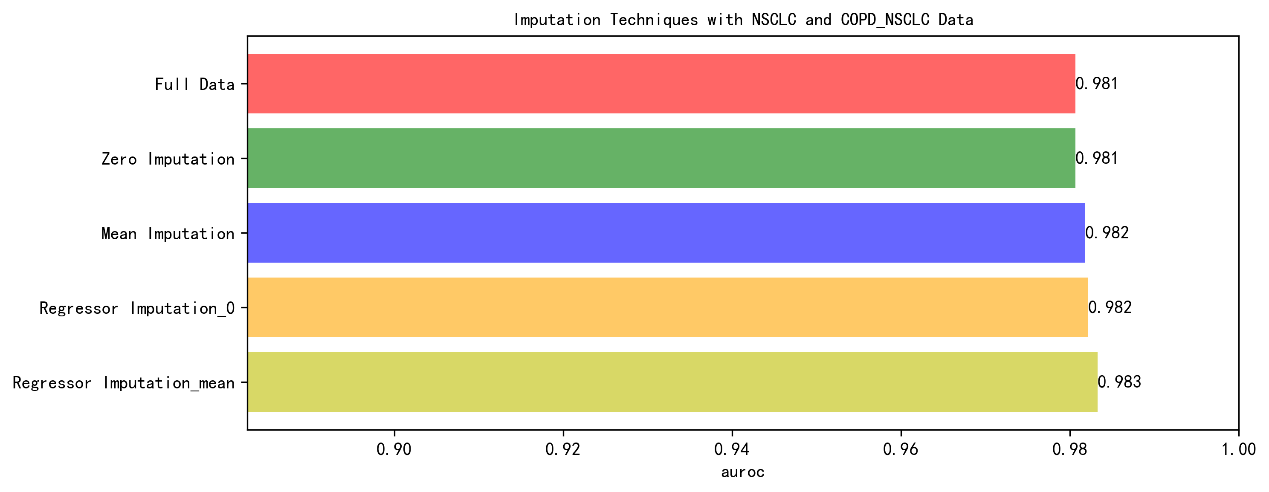 |

**Supplementary Figure S2.** AUROC comparison of different pre-processing.

**Supplementary Table S3.** Hyperparameters of KNN model.

| knn_param_name | knn_param |
| --- | --- |
| columntransformer__num | StandardScaler() |
| kneighborsclassifier__algorithm | auto |
| kneighborsclassifier__n_neighbors | 5 |

**Supplementary Table S4.** Hyperparameters of LR model.

| lr_param_name | lr_param |
| --- | --- |
| columntransformer__num | KBinsDiscretizer(encode='ordinal', n_bins=3, strategy='kmeans') |
| logisticregression__C | 1 |
| logisticregression__penalty | l1 |
| logisticregression__solver | liblinear |

**Supplementary Table S5.** Hyperparameters of XGB model.

| xgb_param_name | xgb_param |
| --- | --- |
| columntransformer__num | KBinsDiscretizer(encode='ordinal', n_bins=3, strategy='kmeans') |
| xgbclassifier__eta | 0.16 |
| xgbclassifier__max_depth | 1 |
| xgbclassifier__min_child_weight | 1 |
| xgbclassifier__n_estimators | 90 |

**Supplementary Table S6.** Hyperparameters of SVM model.

| svm_param_name | svm_param |
| --- | --- |
| columntransformer__num | KBinsDiscretizer(encode='ordinal', n_bins=3, strategy='kmeans') |
| svc__C | 0.1 |
| svc__class_weight | None |
| svc__gamma | 1 |
| svc__kernel | linear |

**Supplementary Table S7.** Hyperparameters of Gaussian Naïve Bayes model.

| gnb_param_name | gnb_param |
| --- | --- |
| columntransformer__num | passthrough |
| gaussiannb__var_smoothing | 1e-8 |

**Supplementary Table S8.** Hyperparameters of Multilayer Perceptron model.

| mlp_param_name | mlp_param |
| --- | --- |
| columntransformer__num | KBinsDiscretizer(encode='ordinal', n_bins=3, strategy='kmeans') |
| mlpclassifier__activation | tanh |
| mlpclassifier__hidden_layer_sizes | (5,5,5) |
| mlpclassifier__max_iter | 20 |
| mlpclassifier__solver | lbfgs |
